# Risk Factors for Atrial Fibrillation in the Dog: A Systematic Review

**DOI:** 10.3390/vetsci11010047

**Published:** 2024-01-21

**Authors:** Giulia Arcuri, Carlotta Valente, Caterina Perini, Carlo Guglielmini

**Affiliations:** Department of Animal Medicine, Production & Health, University of Padova, Viale dell’Università 16, 35020 Legnaro, PD, Italy; giulia.arcuri@phd.unipd.it (G.A.); carlotta.valente@unipd.it (C.V.); caterina.perini.1@studenti.unipd.it (C.P.)

**Keywords:** canine, supraventricular arrhythmia, cardiac disease, electrocardiography, echocardiography

## Abstract

**Simple Summary:**

Atrial fibrillation (AF) is the most common supraventricular arrhythmia in dogs and can lead to a severe decline in the cardiac function. In the last decade, an increased burden of clinical articles has been published that evaluated different aspects of AF in dogs with cardiac disease, particularly in animals affected by myxomatous mitral valve disease (MMVD) and dilated cardiomyopathy (DCM). In this study, we aimed to determine the risk factors for AF in the dog. Therefore, we performed a comprehensive systematic review and critical evaluation of the veterinary literature that reports the risk factors for the development of AF in the dog following the Prisma 2020 guidelines. High bodyweight and left atrial enlargement emerged as predominant risk factors for AF development in dogs with cardiac diseases. Importantly, distinct risk factors for AF were identified between dogs with MMVD and those with DCM (e.g., presence of congestive heart failure in dogs with MMVD but not in those with DCM), highlighting the nuanced nature of AF etiology in different canine cardiac conditions. Furthermore, significant differences in risk factors were observed between dogs and humans. In particular, advanced age and male sex are not reliable indicators of an increased risk of AF in dogs.

**Abstract:**

Different risk factors for atrial fibrillation (AF) development have been identified in numerous studies on humans, but this information is less clearly available on the dog. The aim of this systematic review is to determine the risk factors for AF in the dog. Following the PRISMA 2020 guidelines, we conducted a comprehensive search using the Web of Science and Scopus databases for articles reporting on cases of spontaneously occurring AF in dogs. The level of evidence was assessed using the Evidence Quality Grading System of the National Institute of Health. One thousand forty-three studies were initially identified, and twenty of them were included in this systematic review involving 2,359,275 dogs, of which 4807 showed spontaneously occurring AF. Genetics, for the Irish Wolfhound, increased body weight, and left atrial enlargement were the main risk factors for the development of AF in dogs with different cardiac diseases, particularly myxomatous mitral valve disease (MMVD) and dilated cardiomyopathy (DCM). However, some differences were found between these two cardiac diseases regarding additional risk factors. In particular, the presence of congestive heart failure and echocardiographic evidence of increased left atrial pressure or the presence of right atrial enlargement emerged as risk factors in dogs with MMVD or DCM, respectively. Furthermore, significant differences in risk factors were observed between dogs and humans. In particular, advanced age and male sex are not reliable indicators of an increased risk of AF in dogs.

## 1. Introduction

Atrial fibrillation (AF) stands as the most prevalent supraventricular arrhythmia in dogs, with an estimated prevalence of 0.15% in the general canine population [1]. This prevalence varies significantly among breeds, ranging from 0.04% in Miniature Poodles to 8.9% in Irish Wolfhounds [1,2,3]. Differences also emerge concerning underlying cardiac diseases, with prevalence ranging from 2.7% to around 45% in dogs with myxomatous mitral valve disease (MMVD) and dilated cardiomyopathy (DCM), respectively [4,5,6]. While some dogs may develop AF without an apparent underlying cardiac disease (referred to as primary or lone AF) [3,7], this arrhythmia typically arises secondary to canine cardiac diseases that induce left atrial remodeling [3,7,8]. The most common contributors include MMVD, DCM, and left-sided congenital heart disease (CHD), particularly in their advanced stages [3]. Notably, left atrial enlargement (LAE), and more broadly, a critical left atrial mass, is identified as a pivotal substrate for AF development across different species [3,9]. Consequently, both LAE and body weight (BW) are recognized as significant risk factors for AF development in dogs [10].

In humans, incident AF is associated with various risk factors, encompassing genetics, age, sex, ethnicity, and clinical factors. Cardiac disorders (e.g., hypertension, heart failure, and coronary artery disease) and non-cardiac disorders (e.g., diabetes mellitus and chronic kidney disease) are acknowledged among these factors [11,12,13,14,15]. Risk factors for the development of AF are less well established in dogs. Regarding presentation, while AF in some dogs may exhibit a paroxysmal pattern [16,17] characterized by spontaneous or following intervention termination within 7 days of onset, the predominant presentation involves a persistent (continuously sustained beyond 7 days), long-standing persistent (continuously endured for more than 12 months), or permanent pattern [3,11]. The chronic absence of atrial contraction and the typically rapid ventricular rate accompanying AF contribute not only to diminished ventricular filling and reduced cardiac output, but also to increased left ventricular filling pressure; moreover, over time, structural alterations in the myocardium develop, which further impair cardiac function (tachycardia-induced cardiomyopathy) [3]. Consequently, AF is associated with an elevated risk of cardiovascular mortality, primarily due to congestive heart failure (CHF) and sudden death [3,18,19,20].

Given the unfavorable prognosis linked to the onset of AF in dogs with cardiac disease, a profound comprehension of the associated risk factors becomes paramount. Consequently, the objective of this paper is to provide a thorough systematic review and evaluation of existing literature documenting the risk factors for canine AF. The reported evidence on the factors influencing the development of AF in dogs was subjected to critical analysis, with a parallel comparison to findings reported in human studies.

## 2. Materials and Methods

### 2.1. Stage 1—Search Strategy

To conduct our systematic review, we adhered to the Preferred Reporting Items for Systematic Reviews and Meta-Analysis (PRISMA) 2020 guidelines [21]. Our search focused on full peer-reviewed studies pertaining to dogs with spontaneously occurring AF. Utilizing the online Scopus and Web of Science databases, we systematically examined records from the inceptions of these databases up to October 2023.

The search employed the following key terms and Boolean operators: “atrial fibrillation” OR “supraventricular arrhythmia” AND “dog” OR “canine” AND “risk factor” OR “predictor” OR “susceptibility” OR “cause” OR “influence” OR “prevalence” OR “incidence”.

All identified records from each search were meticulously cataloged in a Microsoft Excel spreadsheet. The data recorded encompassed author names, paper titles, journal names, publication years, volumes, issues, and page numbers. Exclusion criteria involved duplicate studies and those published in non-English languages.

### 2.2. Stage 2—Screening

For the initial screening, two reviewers (GA and CP) independently assessed each retrieved record based on the title and abstract. The objective was to exclude incomplete studies (i.e., records with only the abstract available), non-veterinary or non-clinical studies (specifically, studies involving the experimental induction of AF in dogs), review articles, and studies pertaining to species other than dogs. In instances where disagreements arose between the initial reviewers, resolution was achieved through consultation with a third reviewer (CG).

### 2.3. Stage 3—Eligibility

Following Stage 2, papers deemed eligible were subjected to independent screening by two authors (GA and CP) based on the title, abstract, and full text. Additionally, the reference lists of all papers included in the final selection underwent scrutiny, and citations not captured in the initial literature search were assessed for potential inclusion in the final corpus. The same reviewers conducted a thorough evaluation of the full texts to determine their relevance to the inclusion criteria. Consensus on the inclusion of papers was achieved through discussion, with a third author (CG) consulted in cases of discrepancies.

Records incorporated into the final corpus adhered to the following criteria:

- Peer-reviewed papers in the English language addressing the topic of canine AF, encompassing all types of arrhythmia (i.e., paroxysmal, persistent, or permanent).
- Papers reporting primary research results, including case series, observational cohort and cross-sectional studies, case-control studies, and randomized controlled trials. Literature reviews and single-case reports were excluded.
- Papers reporting the inclusion of dogs diagnosed with AF and conditions associated with the development of AF.

### 2.4. Quality Assessment

To gauge the quality of eligible full-text articles, we employed the Evidence Quality Grading System tool developed in 2013 by the National Heart, Lung, and Blood Institute of the National Institute of Health (NIH) [available at https://www.nhlbi.nih.gov/health-topics/study-quality-assessment-tools, accessed on 17 October 2023]. Two independent reviewers (GA and CP) conducted the quality assessment, and any disparities between reviewers were resolved through consultation with a third reviewer (CG).

In summary, this tool aids in evaluating the strength of evidence in various study types (e.g., observational cohort and cross-sectional studies or case series) through a set of 12 or more questions, each with three possible answers: Yes, No, and Other (i.e., cannot determine, not applicable, or not reported). Studies were classified based on the following criteria:

- High-quality studies (Good): Yes for all criteria.
- Moderate-quality studies (Fair): Yes for most criteria.
- Low-quality studies (Poor): No or Other for most criteria.

## 3. Results

### 3.1. Identification and Selection of Relevant Articles

**Figure 1:**
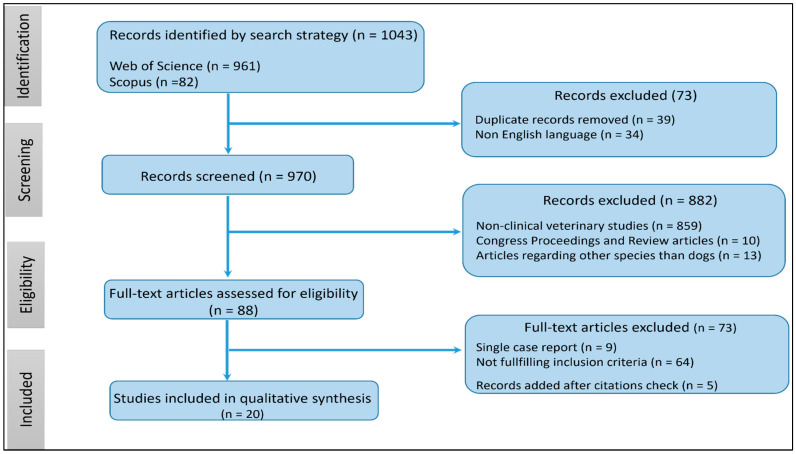
Flowchart of the literature search strategy.

A flow diagram of the search procedure is shown in Figure 1.

A total of 1043 records were initially identified from Scopus (n = 82) and Web of Science (n = 961). Exclusions were made for 39 records due to duplicate citations and 34 records for being in a non-English language. Among the remaining 970 records, 882 were excluded for various reasons: non-clinical veterinary studies (n = 859), incomplete studies, and review articles (n = 10), as well as studies related to species other than dogs (n = 13). Following the assessment of 88 full-text articles for eligibility, 73 records were excluded due to being single-case reports or not reporting conditions associated with the development of AF. Additionally, 5 articles were added after searching for citations not captured in the initial search. Consequently, our systematic review includes 20 studies involving 2,359,275 dogs, of which 4807 had spontaneously occurring AF.

For each paper in the final corpus, the following details were meticulously recorded in a Microsoft Excel spreadsheet:

- Publishing details: First author and year of publication.
- Study details: Study design, overall sample size (total number of dogs included in the study, including controls where used), clinical data, including breed, underlying heart diseases, the percentage of dogs with AF and CHF, and quality rating.
- Study outcomes: Identified risk factors.

### 3.2. Study Characteristics and Quality Assessment

A comprehensive overview of study characteristics and their quality rating is presented in Table 1. Regarding study design, 11 (55%) studies were retrospective case-control, 1 (5%) was retrospective cross-sectional, 2 (10%) were retrospective cohort, 3 (15%) were retrospective observational, and 3 (15%) were case series. The extracted literature spanned a wide timespan from 1971 to 2023, with the majority of articles (14/20 studies, 70%) published since 2016. Most papers (14/20 studies, 70%) included different breeds in their studies, while others focused solely on a single breed, such as Irish Wolfhound (3/20 studies, 15%), Dogue de Bordeaux, or Dobermann Pinscher (1/20 study, 5%, each). One paper did not report the breeds included in the study (1/20 study, 5%).

The most frequently reported underlying cardiac diseases were CHD (8/20 studies, 40%), MMVD (11/20 studies, 55%), DCM (9/20 studies, 45%), and arrhythmogenic right ventricular cardiomyopathy (1/20 study, 5%). In four studies (20%), the precise underlying cardiac disease was not reported.

In the final corpus, no high-quality study was identified. However, 15 articles (75%) demonstrated a moderate reporting quality, while 5 articles (25%) had a low reporting quality. The main reasons for a lower quality rating were often attributed to the lack of justification for sample size and inadequacies in the methodology or an insufficiently detailed reporting of results.

### 3.3. Risk Factor Results

Comprehensive results summarizing the identified risk factors for each paper in this review are detailed in Table 1. Risk factors contributing to the development of AF encompass both clinical and echocardiographic variables. Major risk factors consistently reported across studies include sex, age, BW, breed, and LAE, including both absolute increases in the left atrial diameter (LAD) and the left atrial diameter to aortic root diameter ratio (LA:Ao ratio). Other clinical and echocardiographic risk factors associated with AF include high heart rate at presentation; left ventricular enlargement, involving increases in both the left ventricular diastolic and systolic diameters normalized to BW; right atrial and ventricular enlargement; the presence of CHF; decreased fractional shortening (FS); and increased peak velocity of the mitral E wave (E max). The utilization of advanced echocardiographic techniques, such as tissue Doppler imaging (TDI) and left atrial strain using speckle tracking echocardiography (STE), identified additional echocardiographic parameters like an increase in the time interval from the onset of the P wave on the electrocardiogram to the peak of the A’ wave (PA-TDI) and a decrease in peak atrial longitudinal strain (PALS). Lastly, the presence of neurally mediated syncope events and genetics were also reported as risk factors underscoring the multifaceted nature of AF development.

### 3.4. Direction of Risk

Among the clinical variables assessed as risk factors for AF development, increasing age was identified as a risk factor in four articles (20%). Male dogs had a higher risk of developing AF in six papers (30%). Breed and BW were discussed in 11 articles (55%), highlighting that dogs of large and giant breeds, and consequently with higher BWs, are at a higher risk of developing AF. Increased heart rate at presentation and the presence of CHF were identified as risk factors in two papers each (10%). Genetics, specifically being an Irish Wolfhound, and neurally mediated syncope were reported in one paper each (5%). Regarding echocardiographic variables, LAE was reported as a risk factor in six papers (30%), right atrial enlargement was mentioned in three papers (15%), decreased FS was reported in two papers (10%), right ventricular enlargement, left ventricular enlargement, increased E max and PA-TDI, as well as decreased PALS, were reported in one paper each (5%).

Due to the retrospective design of all selected studies, statistical analysis was limited in many instances, with some studies relying on comparisons between dogs with and without AF or lacking statistical analysis altogether, particularly in case series studies. Specific statistical tests aimed at identifying independent risk factors for developing AF, such as univariate and multivariable logistic regression analyses, were applied in seven articles (35%). Notably, four of these papers (20%) specifically focused on the search for clinical and echocardiographic risk factors, utilizing these tests to possibly estimate the odds ratio (OR) of AF developing in dogs with MMVD and DCM. Furthermore, one paper conducted a heritability analysis on Irish Wolfhounds to determine the genetic contribution to AF in this specific canine breed.

Table 2 presents a comparison between humans and dogs with MMVD or DCM concerning clinical risk factors for developing AF. Unlike humans, advanced age and sex do not represent risk factors for the development of the arrhythmia in dogs, while increased BW and the presence of CHF are recognized risk factors for AF in both humans and dogs with MMVD but not in animals with DCM.

Table 3 summarizes echocardiographic risk factors for AF in dogs with MMVD and DCM, including odds ratios (ORs). Notably, increased absolute LAD represents a demonstrated risk factor for AF in both dogs with MMVD (OR 5.28) and DCM (OR 3.58), while a relative increase in the left atrial dimension expressed by the LA:Ao ratio is a risk factor only for animals with MMVD (OR 14). Additionally, increased E max and decreased FS (OR 2.2 and 0.91, respectively), or the presence of right atrial enlargement (RAE) (OR 4.02), are risk factors for AF but vary between dogs with MMVD or DCM.

Table 4 presents detailed information on echocardiographic risk factors associated with canine AF, including cut-off values, sensitivity, specificity, and overall diagnostic accuracy expressed as the area under the curve (AUC) of the receiver operating characteristic curve, to predict the development of the arrhythmia. In dogs with different cardiac diseases, namely CHD, MMVD, and DCM, the PA-TDI at the cut-off of 81.2 ms demonstrated high accuracy in predicting AF development (AUC = 0.896). Considering individual heart diseases, LAD (AUC = 0.979), the LA:Ao ratio (AUC = 0.931), and E max (AUC = 0.900) at the cut-offs of >3.45 cm, >1.8, and >102 cm/s, respectively, exhibited the highest accuracy in predicting the presence of the arrhythmia in dogs with MMVD. In dogs with DCM, the LAD at the cut-off of >4.66 cm emerged as the best echocardiographic predictor of AF (AUC = 0.816). However, considering the different prevalences of the arrhythmia between the two most common canine-acquired cardiac diseases, an LAD > 3.45 cm and >4.66 cm had positive predictive values of 20.7% and 67.5% in dogs with MMVD and DCM, respectively.

## 4. Discussion

In this systematic review, 20 articles involving a substantial canine population of 2,359,275 dogs, with 4807 cases of AF, were comprehensively evaluated. Notably, no paper received an excellent quality rating, with the majority, particularly those published since 2016, falling into the “fair” category according to established guidelines. Papers from earlier decades, particularly the 1970s and 1980s, demonstrated lower quality ratings and were assessed as “poor”.

While the use of the term “lone” or primary atrial fibrillation (AF) has been questioned in human medicine [11], it persists in the context of canine medicine to denote a condition where AF develops without a recognizable underlying cardiac disease [7]. This phenomenon is observed in a minority of dogs, predominantly among large or giant breeds, with the Irish Wolfhound exhibiting a genetic predisposition for the arrhythmia [29]. Additionally, a subset of dogs has been reported with paroxysmal AF presumed to be neurally mediated, following syncopal episodes [17]. However, in the canine context, AF predominantly takes on a long-standing persistent or permanent nature and is commonly associated with underlying structural cardiac diseases, particularly DCM, MMVD, and CHD. This form of AF is referred to as secondary AF [7].

Numerous studies in human medicine have delved into the multifaceted landscape of AF risk factors [11,12,13,14,15]. These investigations span various domains, encompassing demographic factors such as age, sex, and ethnicity; health behaviors, including smoking, alcohol intake, and physical activity; broader health factors like height, hypertension, obesity, diabetes mellitus, and renal dysfunction; cardiovascular disorders, including heart failure, valvular disease, coronary artery disease, and CHD; and genetic factors [11,12,13,14,15]. This systematic review affirms that certain risk factors implicated in human AF may also contribute to the development of AF in dogs. Notably, cardiovascular disorders, in particular valvular disease, exhibit relevance across both species. However, other factors integral to human AF, such as health behaviors (e.g., smoking tobacco and alcohol intake) and broader health factors (e.g., height, concurrent non-cardiac disorders), are either not applicable or have been inadequately investigated in the canine population [4,5].

In the context of demographic factors, parallels and distinctions emerge between humans and dogs regarding AF. In humans, recognized risk factors for incident AF include genetics, advanced age, male sex, and Caucasian ethnicity [11]. In dogs, many studies have reported a higher prevalence of AF in large-breed animals [1,5,6,9,10,11,16,18,19,20,21,22,23,24]. Notably, a high heritability estimate for AF, with a likely dominant mode of inheritance, has been demonstrated specifically in Irish Wolfhounds [29]. However, genetic studies in other commonly affected canine breeds are currently lacking. The observed higher prevalence of AF in large-breed dogs appears to be linked to their predisposition to DCM rather than a specific genetic predisposition for the arrhythmia [3,5].

Concerning sex, earlier reports lacking specific statistical analyses suggested a higher likelihood of AF in male dogs [1,8,16,17,24,25]. However, recent studies employing rigorous statistical tests, such as multivariable logistic regression models, indicate that male sex is not a direct risk factor for AF in dogs, unlike in humans [4,5,30,31]. The previously reported prevalence of AF in male dogs is likely associated with their predisposition to acquired heart diseases, such as MMVD or DCM [6,33]. Similarly, there have been conflicting findings regarding age as a risk factor for AF in dogs. While some older studies suggested a correlation between age and AF [2,22,24,25], recent investigations utilizing multivariable logistic regression models did not find age to be a significant risk factor in dogs with MMVD and DCM [4,5,31]. Unlike in humans, where aging and age-related underlying disorders are linked to atrial structural remodeling [34], studies on age-related atrial remodeling in dogs with AF are lacking. Convincing evidence demonstrating a true relationship between advanced age and the development of AF in dogs is yet to be established.

Elevated BW emerges as a significant risk factor for the development of AF in both humans and dogs, underscoring a commonality in the pathogenesis [4,8,11,16,18,22,23,24,25,28]. This association is particularly notable in dogs with MMVD [4], a cardiac condition typically affecting small-sized dogs [33]. Intriguingly, in animals with MMVD, those with a BW exceeding 20 kg exhibit a 5.8 times greater risk of developing AF, highlighting the weight-related susceptibility in this canine population [35]. In contrast, in dogs affected by DCM, a cardiac disease typically observed in large or giant breeds but not in small dogs, recent research challenges the notion of BW as an independent risk factor for AF after having conducted multivariable analyses [5]. These findings imply that while BW holds significance as a risk factor for AF in the general canine population with cardiac disease, as evidenced in a study encompassing dogs with either MMVD or DCM [28], its role becomes less pivotal when focusing on a more specific population—specifically, dogs with DCM. In this subset, where a majority possesses a high BW, the impact of BW as a predictor for AF development diminishes.

In close association with BW, left atrial dimension emerges as a pivotal risk factor for AF not only in humans [11] but also across various animal species [3,8,9]. The left atrium, along with the pulmonary veins, assumes a central role in the initiation and perpetuation of AF in humans [34]. Left atrial enlargement serves as a readily identifiable manifestation of left atrial remodeling in response to cardiac disease, as elucidated in five articles within this systematic review [2,4,5,8,26]. The use of two-dimensional echocardiography facilitates the assessment of LAE through both the measurement of absolute LAD and the calculation of the LA:Ao ratio [36]. However, in assessing the prediction of AF development in both dogs with MMVD and DCM, it is evident that LAD stands out as the superior predictor. Notably, cut-offs of >3.45 cm for MMVD and >4.66 cm for DCM have been identified [4,5]. In comparison to the LA:Ao ratio, these echocardiographic parameters demonstrate high sensitivity in predicting AF development. Importantly, the specificity of LAD surpasses that of the LA:Ao ratio. Consequently, the diagnostic accuracy of LAD is notably higher, reflected in the AUC values of 0.979 and 0.816 for dogs with MMVD and DCM, respectively. In contrast, the LA:Ao ratio exhibits lower AUC values of 0.931 and 0.637 for dogs with MMVD and DCM, respectively [4,5]. The assessment of absolute LAD emerges as a more practical predictor of AF development in dogs, contrasting with the commonly favored index of relative LAE, the LA:Ao ratio, preferred by veterinary echocardiographers for assessing left atrial sizes in dogs [36]. It is crucial to note that the observed high diagnostic accuracy of the specified cut-off limits for predicting AF presence should be interpreted in the context of the significantly higher prevalence of the arrhythmia in dogs with DCM [5,6] compared to those with MMVD [4]. For instance, it can be predicted that approximately one in five dogs with MMVD and a LAD > 3.45 cm will develop AF, in contrast to roughly two in three dogs with DCM and a LAD > 4.66 cm [4,5]. Drawing a parallel to human medicine, where left atrial diameter is employed to predict new-onset AF in cases of embolic stroke of unknown origin, patients with a LAD higher than 4.0 cm exhibit a twofold higher risk of developing paroxysmal AF compared to those with a LAD lower or equal to 4.0 cm [37].

Further echocardiographic markers independently predicting the development of AF in dogs were discerned in this systematic review. Specifically, increased mitral E max and decreased FS emerged as predictors in dogs with MMVD [4]. Concurrently, right atrial enlargement was identified as a predictive factor in dogs with DCM [5,30]. The elevation in mitral E max serves as an indirect indicator of heightened left atrial pressure [38]. Consequently, it concurs with progressive left atrial dilatation and remodeling, which act as a substrate for AF initiation and maintenance [39]. It is noteworthy that increased mitral E max also carries negative prognostic implications in dogs with MMVD [40,41,42,43,44]. Additionally, the presence of right atrial enlargement was identified as a correlate of AF development in dogs with DCM [5,30], mirroring findings observed in human patients with heart failure and preserved ejection fraction [45]. Notably, dogs with cardiac disease and AF are more prone to exhibiting signs of right-sided CHF than their counterparts without AF [28]. While considerable attention has been directed toward assessing left atrial structural changes and dysfunction in subjects with AF, the roles of right atrial enlargement and dysfunction remain underexplored in both human and canine studies [28,46]. Further research in this domain is needed. Moreover, parameters such as increased left ventricular diameters and decreased PALS in dogs with MMVD and increased PA-TDI in dogs with left-sided cardiac diseases exhibit potential clinical relevance in predicting AF [4,27,31]. Specifically, a cut-off for the PA-TDI interval at 81.2 ms demonstrated good accuracy in predicting the presence of the arrhythmia [27]. In human medicine, advanced echocardiographic techniques like TDI and STE have proven valuable in evaluating left atrial function [47,48]. In dogs, reduced PALS have been indicative of an increased risk of cardiac death in dogs with MMVD [31].

The roles of heart rate and CHF in dogs with secondary AF are topics marked by controversy. Elevated heart rate is a prevalent finding in dogs with cardiac disease and AF, as it is often associated with increased sympathetic tone, a pathophysiological consequence of advanced cardiac disorders [4,5,26,28,31]. However, the persistent elevation in heart rate linked to AF can potentially contribute to tachycardia-induced cardiomyopathy [2,49]. Understanding the cause–effect mechanism between increased heart rate and AF remains challenging, both in humans and dogs, given the bidirectional relationship. An elevated heart rate is commonly observed as a clinical hallmark in individuals with AF [3,11]. In the context of this systematic review, two papers [26,28] highlighted an elevated heart rate at presentation as a risk factor for the development of AF. One of these studies reported an OR of 1.123, shedding light on the potential significance of heart rate in predicting AF development [28]. The role of heart rate in the prognosis of dogs with AF introduces a notable dimension, where a high heart rate serves as a negative prognostic factor. Dogs with a heart rate below 125 bpm, as recorded via 24 h Holter monitoring, exhibit a longer survival time compared to their counterparts with heart rates exceeding 125 bpm [19,50]. High heart rate, assessed during echocardiographic examination, also represents an independent predictor of negative outcome in dogs with AF associated with MMVD or DCM [51]. Furthermore, the presence of CHF emerges as an independent factor associated with the development of AF in dogs with MMVD [4], mirroring observations in human patients with cardiac disease [11]. However, this association is not replicated in animals with DCM [5]. In a nuanced exploration, a multivariable logistic regression model, encompassing the LAD and FS, revealed that dogs with a present or past history of decompensated MMVD carried a fivefold increased risk of developing AF compared to those with compensated MMVD [4]. The coexistence of AF and CHF is a recurrent theme in both human and canine patients with cardiac disease [4,5,28,52,53]. Yet, understanding the precise cause–effect relationship between these two conditions proves challenging, given their shared pathophysiology and the intricate temporal relationship, particularly in dogs.

This systematic review, while offering valuable insights, is not immune to certain limitations. Firstly, the intricate task of comparing different study populations poses a challenge, particularly when contrasting studies focused solely on a specific breed with those encompassing a diverse canine population. The inherent variability in breeds introduces complexities that warrant cautious interpretation of comparative findings. Secondly, the expansive timespan covered by the literature (1971–2023) introduces potential influences from evolving research methodologies and diagnostic techniques. The inherent differences across this temporal spectrum, especially in older case series studies, where specific statistical evaluations were often lacking, underscore the need for a discerning lens when synthesizing findings. The retrospective design of all included studies, while valuable in identifying associated risk factors for AF development, inherently falls short of establishing causality. Furthermore, it is crucial to note that none of the studies reviewed attained a high-quality classification. This inherent limitation underscores the need for a nuanced interpretation of the systematic review results. While the identified risk factors provide valuable insights, the overall quality rating of the studies should temper the certainty with which these factors are applied in clinical contexts. Prospective longitudinal studies should emerge as a crucial next step to unravel the cause–effect relationships between potential risk factors and the onset of this arrhythmia. Lastly, the search strategy employed, involving the use of Boolean operators “AND” and “OR” across all fields of papers (title, abstract, and full text), aimed to maximize the retrieval of relevant papers. However, despite these efforts, the possibility remains that some papers were inadvertently overlooked. Factors such as the absence of certain papers from the searched databases could contribute to potential omissions. Acknowledging these limitations accentuates the need for ongoing exploration and refinement in future research endeavors.

## 5. Conclusions

Atrial fibrillation emerges as a prevalent complication in canine left-sided cardiac diseases, particularly in DCM. The insights gleaned from this study hold valuable implications for veterinary clinicians. Understanding the identified risk factors provides a foundation for predicting the onset of this prevalent and potentially perilous arrhythmia in canine patients. The primary risk factors for AF development in dogs include high BW and LAE. Importantly, distinct risk factors for AF were identified between dogs with MMVD and those with DCM, highlighting the nuanced nature of AF etiology in different canine cardiac conditions. Notably, the presence of CHF in dogs with MMVD adds a layer to the risk profile, mirroring parallels seen in humans. In contrast to human predictors, advanced age and male sex do not serve as reliable indicators of a heightened risk of AF in dogs. The measurement of the absolute LAD stands out as the optimal echocardiographic predictor for AF in dogs. Moreover, the integration of advanced echocardiographic techniques such as TDI and STE holds promise for clinical utility in forecasting AF development. The current body of literature on AF risk factors in dogs is predominantly retrospective, underscoring the need for meticulously designed prospective studies. Exploring additional predictors, including electrocardiographic parameters, in such studies can significantly contribute to expanding our understanding of canine AF and refining predictive models.

## Figures and Tables

**Table 1:** Summary details of the 20 studies included in the final systematic review evaluating the risk factors for the development of atrial fibrillation in the dog.

First Author (Reference)	Year	Design	Overall Sample Size	Breed	Cardiac Disease	AF (%)	CHF (%)	Risk Factors	Quality Rating
Bolton GR [16]	1971	CaS	5	Various	CHD, MMVD	100	80	Sex; BW	Poor
Bohn FK [22]	1971	R-OB	877	Various	MMVD, CHD, OHD	6.3	90.9	Sex; Age; Breed	Poor
Boevé MH [23]	1984	C	59	Various	CHD, NSHD	100	100	Breed; Sex	Poor
Bonagura JD [24]	1986	CaS	81	Various	DCM, MMVD	100	NR	Breed; Sex; Age	Poor
Guglielmini, C [8]	2000	CC	205	NR	CHD, MMVD, DCM	24.4	NR	BW; LAE (LAD)	Fair
Westling, J [1]	2008	R-OB	2,352,633	Various	NSHD	0.15	NR	Large breed; Sex	Poor
Vazquez, DMP [17]	2016	CaS	7	Various	MMVD, DCM, CHD	100	57.1	Neurally mediated syncope	Fair
Jung, SW [18]	2016	CC	64	Various	MMVD	51.5	100	BW	Fair
Noszczyk-Nowak, A [25]	2017	R-OB	1189	Various	Cardiological referrals	13.4	NR	BW; Age; Sex	Fair
McAulay, G [26]	2018	CC	64	DdB	CHD, Cm, NCm	39	NR	HR; LAE (LA:Ao); LVE; FS; RAE/RVE	Fair
Neves, J [27]	2018	CC	42	Various	CHD, MMVD, DCM	50	NR	PA-TDI	Fair
Vollmar, C [20]	2019	CC	104	IW	Asymptomatic dogs	50	36.5	LAE (LAD)	Fair
Ward, J [28]	2019	CC	220	Various	DCM, MMVD	27.7	100	HR; BW	Fair
Fousse, LS [29]	2019	CC	463	IW	NSHD	70.6	NR	Genetics	Fair
Friederich, J [30]	2020	CC	48	DP	DCM	47.9	100	RAE	Fair
Tyrrell, WD [2])	2020	C	618	IW	DCM	8.9	NR	Age, LAE	Fair
Baron Toaldo, M [31]	2020	CC	44	Various	MMVD	50	77.2	PALS	Fair
Guglielmini, C [4]	2020	CrS	2194	Various	MMVD	2.7	89.8	BW; LAE (LAD, LA:Ao); E max; FS; CHF	Fair
Borgeat, K [32]	2021	CC	269	Various	CHD, MMVD, DCM, ARVC	52.7	66.1	BW; CHF	Fair
Guglielmini, C [5]	2023	CC	89	Various	DCM	43.8	94.8	LAE (LAD); RAE	Fair

Abbreviations: BW = body weight; C = cohort study; CC = case-control study; CHD = congenital heart disease; CHF = congestive heart failure; Cm = cardiac mass; CaS = case series study; CrS = cross-sectional study; DbB = Dog de Bordeaux; DCM = dilated cardiomyopathy; DP = Dobermann Pinscher; E max = peak velocity of the mitral E wave; FS = fractional shortening; IW = Irish Wolfhound; LA:Ao = left atrial diameter to aortic diameter ratio; LAD = left atrial diameter; LAE = left atrial enlargement; LVE = left ventricular enlargement; MMVD = myxomatous mitral valve disease; NCm = non cardiac mass; NR = not reported; NSHD = non specified heart disease; OHD = other heart disease; PALS = peak atrial longitudinal strain; PA-TDI = time interval from the onset of the P wave on electrocardiogram to the peak of the A’ wave; RAE = right atrial enlargement; R-OB= retrospective observational study; RVE = right ventricular enlargement.

**Table 2:** Comparison of risk factors for the development of atrial fibrillation between humans [11] and dogs with myxomatous mitral valve disease (MMVD) [4] or dilated cardiomyopathy (DCM) [5].

Risk Factors	Humans	DOGS	
		MMVD	DCM
Genetics	Yes	NA	Yes (IWH)
Age	Yes	No	No
Male sex	Yes	No	No
Life style ^1^	Yes	NA	NA
Obesity	Yes	NA	NA
Body weight	Yes	Yes	No
Concurrent non-cardiac diseases ^2^	Yes	NA	NA
Congestive heart failure	Yes	Yes	No

^1^ Physical activity, alcohol consumption, and smoking; ^2^ Diabetes, chronic kidney disease, inflammatory diseases, chronic obstructive pulmonary disease, and obstructive sleep apnea; NA: not evaluated/not applicable; IWV: Irish Wolfhound.

**Table 3:** Echocardiographic risk factors of the development of atrial fibrillation and corresponding odds ratio (OR) in dogs with myxomatous mitral valve disease (MMVD) [4] or dilated cardiomyopathy (DCM) [5,30].

Risk Factors	MMVD-OR		DCM-OR	
Left atrial diameter	Yes	5.28	Yes	3.58
Left atrial diameter to aortic diameter ratio	Yes	14	No	-
Right atrial enlargement	NE	-	Yes	4.02
Peak velocity of mitral E wave	Yes	2.2	No	-
Fractional shortening	Yes	0.91	No	-

NE: not evaluated.

**Table 4:** Diagnostic accuracy, sensitivity (Se), and specificity (Sp) of clinical and echocardiographic variables for predicting the development of atrial fibrillation in dogs with myxomatous mitral valve disease (MMVD) or dilated cardiomyopathy (DCM).

Variable	Cardiac Disease	Cut-Off	AUC	Se (%)	Sp (%)	Reference
Body weight (kg)	MMVD	7.6	0.735	96.6	44.4	[4]
	DCM	>36	0.740	79	58	[5]
LAD (cm)	MMVD	>3.45	0.979	98.3	89.8	[4]
	DCM	>4.66	0.816	90	66	[5]
LA:Ao	MMVD	>1.8	0.931	98.3	78.5	[4]
	DCM	>1.73	0.637	95	38	[5]
Ao	DCM	>2.3	0.686	85	50	[5]
LVDDn	MMVD	>1.82	0.854	81.4	82.2	[4]
LVSDn	MMVD	>1.08	0.875	79.7	86.7	[4]
FS (%)	MMVD	≤40.1	0.682	70.7	60.1	[4]
E max (cm/s)	MMVD	>102	0.900	91.1	79.0	[4]
	DCM	>79	0.647	89	42	[5]
PA-TDI (ms)	CHD, MMVD, DCM	81.2	0.896	81	90.5	[27]
PALS (%)	MMVD	≤28	0.721	80	65	[31]

Abbreviations: Ao = aortic diameter; AUC = area under the curve; E max = peak velocity of the mitral E wave; FS = fractional shortening; LA:Ao = left atrial diameter to aortic diameter ratio; LAD = left atrial diameter; LVDDn = left ventricular diastolic diameter normalized to body weight; LVSDn = left ventricular systolic diameter normalized to body weight; PALS = peak atrial longitudinal strain; PA-TDI = time interval from the onset of the P wave on electrocardiogram to the peak of the A’ wave.

## Data Availability

Newly generated data (reanalyzed from original work) are contained within the article.
